# Machine Learning: A Multicenter Study on Predicting Lateral Lymph Node Metastasis in cN0 Papillary Thyroid Carcinoma

**DOI:** 10.1210/clinem/dgaf070

**Published:** 2025-02-08

**Authors:** Jing Zhou, Daxue Li, Jiahui Ren, Chun Huang, Shiying Yang, Mingyao Chen, Zhaoyu Wan, Jinhang He, Yuchen Zhuang, Song Xue, Lin Chun, Xinliang Su

**Affiliations:** Department of Thyroid and Breast Surgery, The First Affiliated Hospital of Chongqing Medical University, Chongqing 40016, China; Department of Breast and Thyroid, Women and Children's Hospital of Chongqing Medical University: Chongqing Health Center for Women and Children, Chongqing 401120, China; Department of Thyroid and Breast Surgery, The First Affiliated Hospital of Chongqing Medical University, Chongqing 40016, China; Department of Breast and Thyroid, Women and Children's Hospital of Chongqing Medical University: Chongqing Health Center for Women and Children, Chongqing 401120, China; Department of Breast and Thyroid, Women and Children's Hospital of Chongqing Medical University: Chongqing Health Center for Women and Children, Chongqing 401120, China; Department of Thyroid and Breast Surgery, The First Affiliated Hospital of Chongqing Medical University, Chongqing 40016, China; Department of Thyroid and Breast Surgery, The First Affiliated Hospital of Chongqing Medical University, Chongqing 40016, China; Department of Thyroid and Breast Surgery, The First Affiliated Hospital of Chongqing Medical University, Chongqing 40016, China; Department of Thyroid and Breast Surgery, The First Affiliated Hospital of Chongqing Medical University, Chongqing 40016, China; Department of Thyroid and Breast Surgery, The First Affiliated Hospital of Chongqing Medical University, Chongqing 40016, China; Department of Breast and Thyroid, Women and Children's Hospital of Chongqing Medical University: Chongqing Health Center for Women and Children, Chongqing 401120, China; Intelligent Integrated Circuits and Systems Laboratory, University of Electronic Science and Technology of China, Chengdu 611730, China; Department of Thyroid and Breast Surgery, Guangyuan Central Hospital, Gungyuan 628017, China; Department of Thyroid and Breast Surgery, The First Affiliated Hospital of Chongqing Medical University, Chongqing 40016, China

**Keywords:** papillary thyroid carcinoma, PTC, clinical lymph node-Negative, cN0, machine learning, ML, prediction model, lateral lymph node metastasis, LLNM

## Abstract

**Background:**

The necessity of prophylactic lateral neck dissection for cN0 papillary thyroid carcinoma (PTC) remains debated. This study aimed to compare traditional nomograms with machine learning (ML) models for predicting ipsilateral lateral and level II, III, and IV lymph node metastasis (LNM).

**Methods:**

Data from 1616 PTC patients diagnosed via fine-needle aspiration biopsy from hospital A were split into training and testing sets (7:3). Two hundred forty-three patients from hospital B served as a validation set. Four dependent variables—ipsilateral lateral and level II, III, and IV LNM—were analyzed. Eight ML models [logistic regression, decision tree, random forest (RF), gradient boosting, support vector machine, K-nearest neighbor, Gaussian naive Bayes, neural networks] were developed and validated using 10-fold cross-validation and grid search hyperparameter tuning. Models were assessed using 11 metrics including accuracy, area under the curve (AUC), specificity, and sensitivity. The best was compared with nomograms using the probability-based ranking model approach (PMRA).

**Results:**

RF outperformed other approaches achieving accuracy, AUC, specificity, and sensitivity of 0.773/0.728, 0.858/0.799, 0.984/0.935, 0.757/0.807 in the testing/validation sets, respectively, for ipsilateral LLNM. A streamlined model based on the top 10 contributing features that includes ipsilateral central lymph node metastasis rate, extrathyroidal extension, and ipsilateral central lymph node metastasis number retained strong performance and clearly surpassed a traditional nomogram approach based on multiple metrics and PMRA analysis. Similar results were obtained for the other dependent variables, with the RF models relying on distinct but overlapping sets of features. Clinical tool implementation is facilitated via a web-based calculator for each of the 4 dependent variables.

**Conclusion:**

ML, especially RF, reliably predicts lateral LNM in cN0 PTC patients, outperforming traditional nomograms.

With advancements in diagnostic techniques like high-resolution ultrasound, ultrasound-guided fine-needle aspiration, and intraoperative frozen section techniques, the incidence of papillary thyroid carcinoma (PTC) has been steadily increasing (1, 2). PTC generally has a favorable prognosis but is frequently accompanied by lymph node metastasis (LNM), occurring in about 20% to 80% of cases (3, 4). LNM significantly impacts the staging and prognosis of PTC patients. Thorough lymph node dissection (LND) plays a crucial role in accurately determining the stage of the disease, guiding subsequent treatment decisions, and ultimately improving patient outcomes.

Therapeutic cervical LND is widely accepted for PTC patients with clinically positive lymph nodes (cN1) (5, 6). However, the use of prophylactic LND for patients with clinically negative lymph nodes (cN0) remains controversial (7-9). According to the 2015 American Thyroid Association guideline recommendations (5), Therapeutic cervical LND is recommended for PTC patients with cN1 status. However, the use of prophylactic central LND is limited to high-risk PTC patients, specifically those with T3 or T4 stage disease. The American Thyroid Association guidelines do not provide explicit recommendations for prophylactic lateral LND. Prophylactic cervical LND is a complex issue with potential benefits and risks, and balancing the surgical advantages and potential complications poses a significant challenge for clinicians. The key to deciding whether to perform prophylactic LND lies in assessing the status of lymph node metastasis (5).

Preoperative evaluation typically includes high-resolution ultrasound and computed tomography. However, the sensitivity of ultrasound in detecting central LNM is only 23% to 41%, and computed tomography provides similar sensitivity, ranging from 38% to 41% (10-12).Occult metastasis, undetected by preoperative imaging, is confirmed in 26% to 56% of cases during postoperative pathology (13-15). This underscores the limitations of relying solely on ultrasound for cN0 patients and highlights the risk of missing optimal surgical opportunities for LNM, which could result in delayed detection and the need for secondary surgeries, negatively affecting patient outcomes.

Intraoperative frozen section analysis is a rapid diagnostic procedure that provides real-time pathological results during surgery, allowing surgeons to assess LNM, particularly central LNM. This information helps surgeons make decisions and determine the appropriate surgical scope during the procedure, thereby avoiding unnecessary secondary surgeries. Intraoperative frozen section analysis is 80% to 90% accurate in identifying central LNM in thyroid cancer patients (16, 17), which helps to compensate for the limitations of preoperative lymph node diagnosis. Furthermore, LNM in PTC follows a predictable pattern. It spreads primarily to the prelaryngeal, pretracheal, and paratracheal lymph nodes. Subsequently, LNM involves the ipsilateral jugular chain and supraclavicular region and ultimately affects the lateral and contralateral cervical regions (18-20). Some researchers suggest that central LNM, including prelaryngeal lymph nodes, may act as sentinel lymph nodes for lateral LNM. This hypothesis could help predict lateral LNM, providing valuable information for clinical decision-making (9). Therefore, performing intraoperative frozen section analysis of various subregions within the central compartment can help predict the presence of lateral LNM and guide surgical strategies.

The rise of artificial intelligence, especially machine learning (ML), offers new opportunities to improve predictive models. ML mitigates the black box issue associated with linear models (19, 21-23). It effectively handles medical big data by standardizing various types of data—whether categorical, continuous, or noncontinuous—thereby mitigating selection bias and omissions. By leveraging ML and currently accessible clinical factors, accurate predictive models can be developed. Our goal is to develop an ML model to accurately predict the presence of cervical lymph node metastasis to facilitate personalized decision-making for prophylactic LND. This approach would facilitate the identification and subsequent removal of hidden metastatic lymph nodes, ensuring accurate diagnosis of occult LNM while minimizing complications from unnecessary dissections. In summary, prophylactic cervical LND is necessary but not for all lymph node regions. It is important to identify the variables that influence the likelihood of lateral LNM. If we can establish a predictive model using preoperative and intraoperative variables, we can improve the sensitivity of preoperative and intraoperative diagnosis and precise selection of good candidates for prophylactic lateral LND. Clinical tool implementation through web-based calculators may enable accurate preoperative and intraoperative predictions of lateral LNM, thereby guiding clinicians for selection of patients most likely to benefit from lateral LND.

## Materials and Methods

### Patient Inclusion and Data Processing

**Figure 1.**
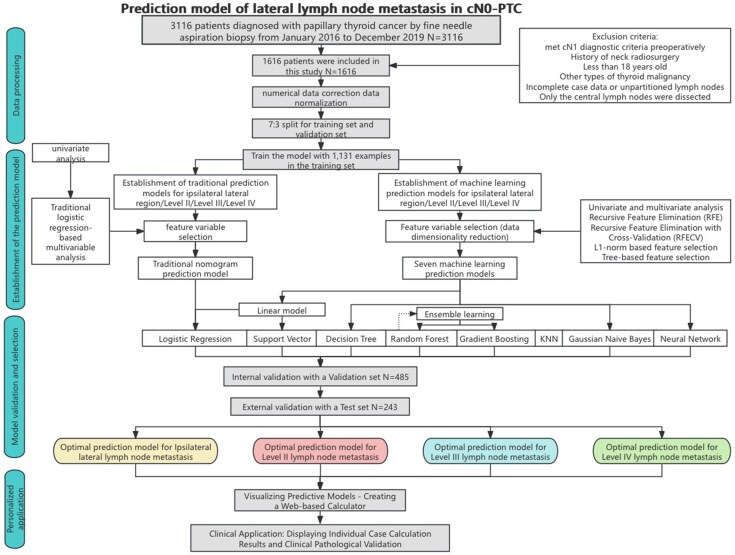
Flowchart of the lateral lymph node metastasis prediction model in cN0 papillary thyroid carcinoma.

This retrospective study, approved by the Ethics Committee of the First Affiliated Hospital of Chongqing Medical University (approval no: 2020-181), included clinical case records of 3116 patients with papillary thyroid carcinoma (PTC) treated at the First Affiliated Hospital of Chongqing Medical University (Hospital A) from 2016 to 2019. Informed consent was obtained from all participants prior to their inclusion in the study. Additionally, the clinical and pathological data of 651 PTC patients treated at the Women and Children's Hospital affiliated with Chongqing Medical University (hospital B) from 2018 to 2020 were collected. The inclusion criteria were: patients of age >18 years with preoperative, biopsy-confirmed (fine-needle aspiration) diagnosis of PTC and radiologically confirmed cN0 status that underwent both central and lateral LND and for whom complete clinical, ultrasound, and pathological data were available (5). Exclusion criteria included a history of neck surgery or radiation therapy or diagnosis of other types of malignant thyroid tumors. After strict application of these criteria (Fig. 1), 1616 patients from hospital A were randomly assigned on a 7:3 ratio to the training and test sets. The external validation set consisted of 243 patients from hospital B. The data from each of the sets was preprocessed as follows: (1) for missing values, we employed mode imputation and mean imputation for categorical and continuous numerical variables respectively. (2) For categorical variables, the optimal cut-off values were calculated for age and tumor diameter. Since the LNM proportion and quantity followed a nonnormal distribution, no optimal cut-off values were applied, and these variables were included as continuous numerical variables in the models. (3) Numerical data correction and normalization were performed to improve the data quality, reduce interfeature differences, and enhance the performance of the ML models. (4) The postoperative pathological results served as the gold standard for determining the presence of ipsilateral lateral LNM (Fig. 1).

### Feature Extraction

#### Selection of conventional feature variables

For clinical features there were 3 variables. Age was defined using an optimal cut-off point of 43 years based on receiver operating characteristic (ROC) curve analysis. Body mass index (BMI) was classified based on Chinese and World Health Organization standards: BMI <18.5 (underweight), 18.5 ≤ BMI < 24 (normal weight), BMI ≥ 24 (overweight).

For ultrasound features there were 9 variables. Parameters included tumor border (extreme invasion, irregular shape/sharpness, smooth/borderless); calcification (no/large comet tail, coarse calcification, peripheral calcification, microcalcification); tumor internal vascularization; tumor peripheral blood flow; location (nonupper, upper); and aspect ratio, composition, internal echo pattern, internal echo homogeneous.

For pathological features there were 6 variables. Tumor size (defined as the maximum diameter of the tumor), extrathyroidal extension (ETE) was defined as tumor invasion beyond the thyroid capsule, including microscopic and gross (maximum ETE, involving the trachea, larynx, and recurrent laryngeal nerve) invasion. ETE was assessed via intraoperative frozen section evaluation. For Hashimoto's thyroiditis, the diagnosis was based on any of the following criteria: (1) thyroid peroxidase antibody level > 50 IU/mL, (2) diffuse heterogeneity on ultrasound, or (3) diffuse lymphocytic thyroiditis on histopathological examination. Side position and multifocality was confirmed by ultrasound and an intraoperative frozen section. Tumor stage was determined by intraoperative frozen pathology.

#### Surgical methods and extraction of intraoperative frozen pathology feature variables

Surgical procedures for PTC at our center (hospital A) included unilateral lobectomy and ipsilateral central LND for tumors <4 cm. In cases of central LNM confirmed intraoperatively, total thyroidectomy and lateral LND were performed. Postlobectomy specimens were divided into prelaryngeal, pretracheal, paratracheal, and recurrent laryngeal nerve nodes, which were sequentially dissected and labeled and then immediately sent for intraoperative frozen section analysis. Postoperative histopathological examination of all resected samples was independently conducted by three pathologists.

Intraoperative frozen pathology feature variables (15 variables), namely, ipsilateral central LNM, prelaryngeal LNM, pretracheal LNM, paratracheal LNM, and recurrent laryngeal nerve LNM, are binary variables. The ipsilateral central lymph node metastasis number (LNMN), prelaryngeal LNMN, pretracheal LNMN, paratracheal LNMN, and recurrent laryngeal nerve LNMN are continuous variables, encompassing both micrometastasis and macrometastasis. The ipsilateral central lymph node metastasis rate (LNMR), prelaryngeal LNMR, pretracheal LNMR, paratracheal LNMR, and recurrent laryngeal nerve LNMR are numerical variables that are calculated as the number of metastatic lymph nodes in a region divided by the number of lymph nodes dissected in that region. Dependent variables, which included ipsilateral lateral LNM and LNM levels (II, III, IV), are binary variables determined on the basis of the LNM status obtained from the final paraffin sections.

As this study was retrospective, the ultrasound and pathological images, particularly intraoperative frozen sections, are frequently either unavailable or unclear, which precluded independent feature extraction from the original images. Therefore, all imaging and pathological features were extracted from careful analysis of ultrasound and pathology reports by 2 professional surgeons with over 5 years of experience.

All the data were manually annotated and standardized according to the aforementioned requirements and then verified by a separate expert (a professional surgeon with over ten years of experience).

### Construction of a Traditional Nomogram Predictive Model

Previous studies have utilized a nomogram-based approach for predicting ipsilateral lateral LNM (18, 19). For comparison purposes, we constructed a nomogram model as follows: initially, a univariate analysis of variance was conducted to screen feature variables, excluding poorly correlated variables. The feature variables with a univariate analysis correlation coefficient *P*-value >.05 were included in the multivariate analysis. Multivariate analysis was performed via logistic regression analysis, and a forest plot was drawn. Multicollinearity was handled by calculating variance inflation factors (> 5 excluded). Independent risk factors were incorporated into a nomogram, The nomogram was constructed by using the training dataset. The goodness-of-fit was assessed using the Hosmer–Lemeshow method, displayed in a calibration curve. Nomogram evaluation metrics were compared with ML models.

### Establishment, Optimization, and Selection of ML Predictive Models

ML model development involved 4 steps:

1. Feature selection: we included 35 feature variables, with age and sex as continuous and categorical variables, respectively. We employed dimensionality reduction techniques to retain the most predictive features: using 6 ML algorithms for feature selection: removing low variance features, univariate feature selection, recursive feature elimination, cross-validated recursive feature elimination, L1-based feature selection, and tree-based feature selection. The authors employed these 6 feature selection methods and compared their performance to identify the best-performing method for variable selection. The selected variables from these methods are shown in Supplementary Material 2. These variables were then integrated into 8 ML models. By plotting the ROC curves and decision curve analysis (DCA) curves of these models, we identified the model with the highest area under the curve (AUC) performance of both the training and testing datasets as the optimal predictive model, determining the final set of features.
2. Model training and optimization: the selected feature variables were incorporated into 8 ML prediction models: Logistic regression, decision tree, random forest (RF), gradient boosting, support vector machine, K-nearest neighbor, Gaussian naive Bayes (GNB), and neural network. Models were developed for ipsilateral lateral LNM and LNM levels (II, III, IV) using the training set data. Tenfold cross-validation and grid search were used for hyperparameter tuning.
3. Model evaluation: we used 11 metrics to assess the performance of 8 ML models. The primary metrics included accuracy, AUC, specificity, and sensitivity, while the other 7 metrics served as supplementary indicators to provide a more comprehensive evaluation. Five types of evaluation plots were generated—ROC curves, DCA curves, calibration curves, precision‒recall curves, and learning curves—to comprehensively identify the optimal model.
4. Model validation and selection: the same evaluation metrics and plots were generated for the test set and validation set to identify the best-performing model.

### Comparison Between the Traditional Nomogram Predictive Model and the Optimal Model Selected by ML

Eleven evaluation metrics, along with ROC, DCA, and learning curves, were used to compare the performance of the optimal ML model and the traditional nomogram. The comparison provided insights into model accuracy, calibration, generalization, and limitations. Additionally, the probability-based ranking model approach (PMRA) and Wald test *P*-values were also used to compare model performance (24). This provided further insight into the relative performance of each model, including the optimal ML model, in accurately predicting outcomes.

### Visualization of the Optimal Model and Clinical Application

The optimal models for predicting ipsilateral lateral LNM and LNM levels (II, III, IV) were identified through comprehensive evaluations. A correlation heatmap was plotted to examine the relationships between feature variables within the predictive model. Feature importance was calculated to determine each variable's percentage contribution to the predictive model, and these were ranked in descending order, visualized with a bubble chart. To ensure the web-based calculator's practicality and simplicity, variables with low importance values (contribution less than 0.05) were excluded from the calculator. This calculator was designed with a user-friendly interface to visualize and apply these predictive models in the clinical setting, thereby enhancing their practical utility. We compared optimal models with different numbers of variables to our traditional nomogram model. Using the PMRA ranking method (24), we identified the minimum set of variables with the highest predictive value, represented in a bar chart ranked by importance and contribution. These refined features were incorporated into a web-based calculator.

### Statistical Analysis

The statistical analysis involved the chi-square test for analyzing binary count data, unordered multiple categorical count data, and ordered multiple categorical count data. Binary logistic regression analysis was used to determine the independent risk factors for ipsilateral lateral LNM and LNM levels (II, III, IV). On the basis of the statistically significant indicators from the binary logistic regression analysis, a nomogram was constructed to create predictive models. The statistical analysis was performed via R Studio, with R version 4.3.2 (R Project for Statistical Computing). The “pROC” package was used to calculate the optimal cut-off values for age and tumor diameter for categorical variables. The “foreign” and “rms” packages were used to create the nomogram predictive models and establish validation curves.

The Python programming language (version 3.11.5; Python Software Foundation, Wilmington, DE, USA) was used for variable selection, model training, and evaluation. For the ML models, the scikit-learn Python library (version 0.24) was used to create models and adjust hyperparameters in Python. Performance metrics for evaluating classification performance were also calculated via the scikit-learn Python library (version 0.24). The design code that was generated via R and Python software and used in this study can be obtained from GitHub (https://github.com/ZJ573693/ML-for-ILLNM).

## Results

### Clinical and Ultrasonographic Pathological Characteristics of the Patients

The training set comprised 302 males and 829 females, with an average age of 42 ± 11.6 years and an average tumor size of 14.3 ± 9.3 mm. The testing set consisted of 132 males and 353 females, with an average age of 41.7 ± 11.9 years and a mean tumor size of 13.9 ± 8.8 mm. The validation set was composed of 17 males and 226 females, with an average age of 36.9 ± 10 years and a tumor size of 13.9 ± 8.8 mm. There were no significant differences in 35 clinical or ultrasonographic pathological characteristics across the 3 sets, ensuring a well-balanced dataset (*P* > .05). Detailed baseline characteristics can be found in Table 1 in Supplementary Material 1 (25). Across the 3 datasets 1859 cN0-PTC patients were included. The overall rates of ipsilateral lateral LNM and LNM levels (II, III, IV) in the combined training, test, and validation sets were 45.8%, 21.3%, 32.5%, and 25.1%, respectively (Table 1). Analysis for each of the sets can be found in Supplementary Table 2 in Supplementary Material 1 (25).

**Table 1.** Univariate analysis of characteristic variables across ipsilateral lateral, level II, level III, and level IV lymph node metastasis status in the combined 1859 patients from the training, test, and validation sets

Characteristic variables	Ipsilateral lateral lymph node metastasis		Level II lymph node metastasis		Level III lymph node metastasis		Level IV lymph node metastasis	
	n = 851 (45.8)	*P*-values	n = 350 (21.3)	*P-*values	n = 605 (32.5)	*P-*values	n = 468 (25.1)	*P*-values
Age > 43, y	328 (38.5)	<.001	140 (40)	.037	219 (36.2)	<.001	176 (37.6)	.002
Male	233 (27.4)	.005	105 (30)	.021	172 (28.4)	.004	121 (25.9)	.385
Classification of BMI								
Underweight	53 (6.3)	.072	22 (6.3)	.159	39 (6.5)	.127	31 (6.6)	.244
Normal	544 (63.9)		214 (61.1)		385 (63.6)		302 (64.5)	
Overweight	254 (29.9)		114 (32.6)		181 (30)		28.8)	
Tumor border								
Smooth or borderless	100 (11.8)	<.001	37 (10.6)	<.001	62 (10.2)	<.001	44 (9.4)	<.001
Irregular-shape or L-shaped	464 (54.5)		175 (50)		322 (53.2)		246 (52.6)	
Extrandular invasion	287 (33.7)		138 (39.4)		221 (36.5)		178 (38)	
Calcification								
No or large comet tail	192 (22.6)	<.001	64 (18.3)	<.001	126 (20.8)	<.001	96 (20.5)	<.001
Coarse calcification	47 (5.5)		21 (6)		35 (5.8)		20 (4.3)	
Microcalcification	603 (70.9)		262 (74.9)		436 (72.1)		347 (74.1)	
Peripheral calcification	9 (1.1)		3 (0.9)		8 (1.3)		5 (1.1)	
Tumor internal vascularization	464 (54.5)	<.001	214 (61.1)	<.001	343 (56.7)	<.001	277 (59.2)	<.001
Tumor peripheral blood flow	458 (53.8)	<.001	221 (63.1)	<.001	339 (56)	<.001	270 (57.7)	<.001
Size > 10.5, mm	572 (67.2)	<.001	249 (71.1)	<.001	408 (67.4)	<.001	318 (68)	<.001
Location, upper	229 (26.9)	.003	146 (41.7)	<.001	151 (25)	.395	92 (19.7)	.022
Mulifocality	586 (68.9)	<.001	236 (67.4)	.005	411 (68)	.001	304 (65)	<.001
Hashimoto's thyroiditis	640 (75.2)	.345	270 (77.1)	.135	452 (74.7)	.296	345 (73.7)	.149
ETE	523 (61.6)	<.001	194 (55.4)	<.001	353 (58.3)	<.001	265 (56.7)	<.001
T staging								
1	419 (49.2)	<.001	144 (41.1)	<.001	278 (46)	<.001	213 (45.5)	<.001
2	106 (12.5)		45 (12.9)		76 (12.6)		50 (10.7)	
3	255 (30)		125 (35.7)		195 (32.2)		158 (33.8)	
4	71 (8.3)		36 (10.3)		56 (9.3)		47 (10)	
Ipsilateral central LNM (+)	788 (92.6)	<.001	323 (92.3)	<.001	568 (93.9)	<.001	447 (95.5)	<.001
Prelaryngeal LNM (+)	339 (39.8)	<.001	203 (58)	<.001	245 (40.5)	<.001	184 (39.3)	<.001
Pretracheal LNM (+)	586 (68.9)	<.001	250 (71.4)	<.001	449 (74.2)	<.001	342 (73)	<.001
Paratracheal LNM (+)	611 (71.8)	<.001	234 (66.9)	<.001	460 (76)	<.001	383 (81.8)	<.001
Recurrent laryngeal nerve LNM (+)	215 (25.3)	<.001	91 (26)	<.001	179 (29.6)	<.001	142 (30.3)	<.001

Data are n (% in each column). *P*-values for between-group comparisons are reported.

Abbreviations: BMI, body mass index; ETE, extrathyroidal extension; LNM, lymph node metastasis.

**Table 2.** Evaluation metrics of the traditional nomogram prediction model in test and validation sets for ipsilateral lateral, level II, level III, and level IV lymph node metastasis

Regions	Accuracy	AUC	Specificity	Sensitivity	NPV	PPV	F1 Score	FPR	Lift	Brier score	Kappa
Testing set (internal validation set)											
Ipsilateral lateral LNM	0.746	0.83	0.752	0.74	0.726	0.765	0.752	0.248	0.962	0.162	0.511
Level II LNM	0.81	0.818	0.284	0.966	0.711	0.82	0.887	0.716	0.366	0.116	0.191
Level III LNM	0.758	0.822	0.536	0.888	0.738	0.765	0.822	0.464	0.75	0.149	0.489
Level IV LNM	0.761	0.779	0.458	0.858	0.508	0.832	0.845	0.542	0.714	0.134	0.322
Validation set (external validation set)											
Ipsilateral lateral LNM	0.685	0.766	0.783	0.588	0.65	0.735	0.654	0.217	1.109	0.196	0.4
Level II LNM	0.787	0.788	0.286	0.938	0.583	0.813	0.871	0.714	0.46	0.137	0.202
Level III LNM	0.659	0.731	0.456	0.781	0.554	0.706	0.742	0.544	0.77	0.194	0.264
Level IV LNM	0.659	0.669	0.333	0.786	0.378	0.752	0.769	0.667	0.967	0.188	0.159

Abbreviations: AUC, area under the curve; FPR, false positive rate; LNM, lymph node metastasis; NPV, negative predictive value; PPV, positive predictive value.

### Construction of Traditional Nomogram Prediction for Ipsilateral Lateral, Level II, Level III, and Level IV LNM

**Figure 2.**
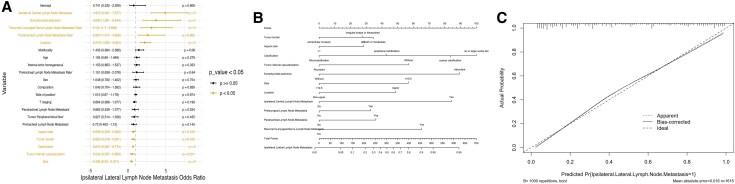
Construction of the nomogram prediction model for ipsilateral lateral lymph node metastasis. (A) Forest plot. (B) Nomogram of the logistic regression model for predicting ipsilateral lateral lymph node metastasis. (C) Prediction model calibration curve.

The independent risk factors for ipsilateral lateral LNM, ranked by the odds ratio (OR) from highest to lowest were as follows: ipsilateral central LNM, ETE, recurrent laryngeal nerve LNMR, paratracheal LNMR, location, aspect ratio, tumor border, calcification, tumor internal vascularization, and tumor size (Fig. 2A). Similarly, the multivariate analyses for LNM levels (II, III, IV) are presented in the forest plots [Supplementary Fig. 1 in Supplementary Material 1 (25)]. A predictive nomogram for ipsilateral lateral LNM was constructed (Fig. 2B), showing good calibration when applied to the validation set (Fig. 2C). Corresponding nomograms for the other dependent variables can be found in Supplementary Fig. 1 of Supplementary Material 1 (25).

### Establishment and Evaluation of the ML Predictive Models

**Figure 3.**
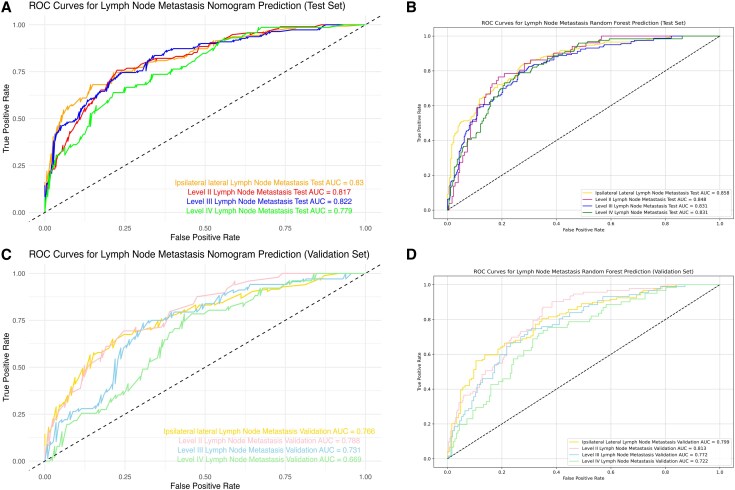
Comparison of the ROC curves for the traditional nomogram prediction model and the optimal machine learning model (random forest) in predicting lateral lymph node metastasis. (A) ROC curve of the nomogram prediction model for ipsilateral lateral lymph node metastasis (levels II, III, and IV) in the test set. (B) ROC curve of the random forest model for ipsilateral lateral lymph node metastasis (levels II, III, and IV) in the test set. (C) ROC curve of the nomogram prediction model for ipsilateral lateral lymph node metastasis (levels II, III, and IV) in the validation set. (D) ROC curve of the random forest model for ipsilateral lateral lymph node metastasis (levels II, III, and IV) in the validation set. Abbreviations: ROC, receiver operating characteristic.

**Figure 4.**
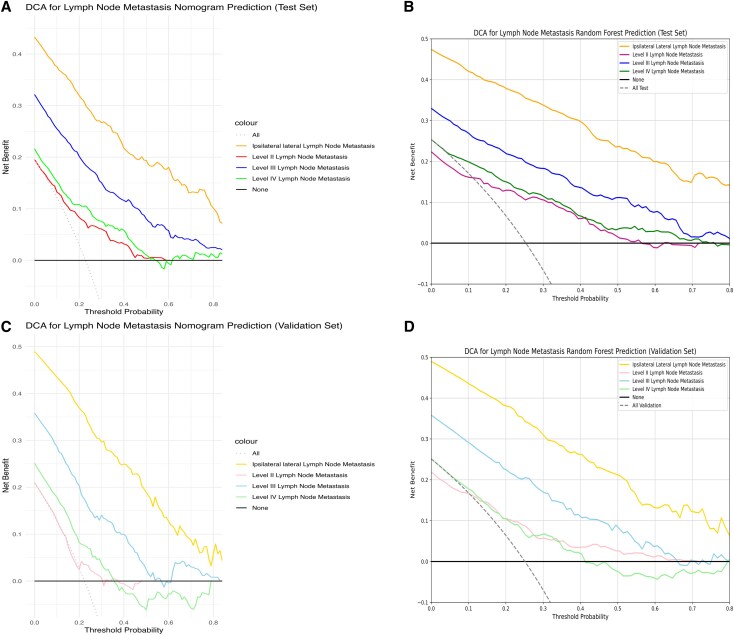
Comparison of the DCA curves for the traditional nomogram prediction model and the optimal machine learning model (random forest) in predicting lateral lymph node metastasis. (A) DCA curve of the nomogram prediction model for ipsilateral lateral lymph node metastasis (levels II, III, and IV) in the test set. (B) DCA curve of the random forest model for ipsilateral lateral lymph node metastasis (levels II, III, and IV) in the test set. (C) DCA curve of the nomogram prediction model for ipsilateral lateral lymph node metastasis (levels II, III, and IV) in the validation set. (D) DCA curve of the random forest model for ipsilateral lateral lymph node metastasis (levels II, III, and IV) in the validation set. Abbreviations: DCA, decision curve analysis.

**Figure 5.**
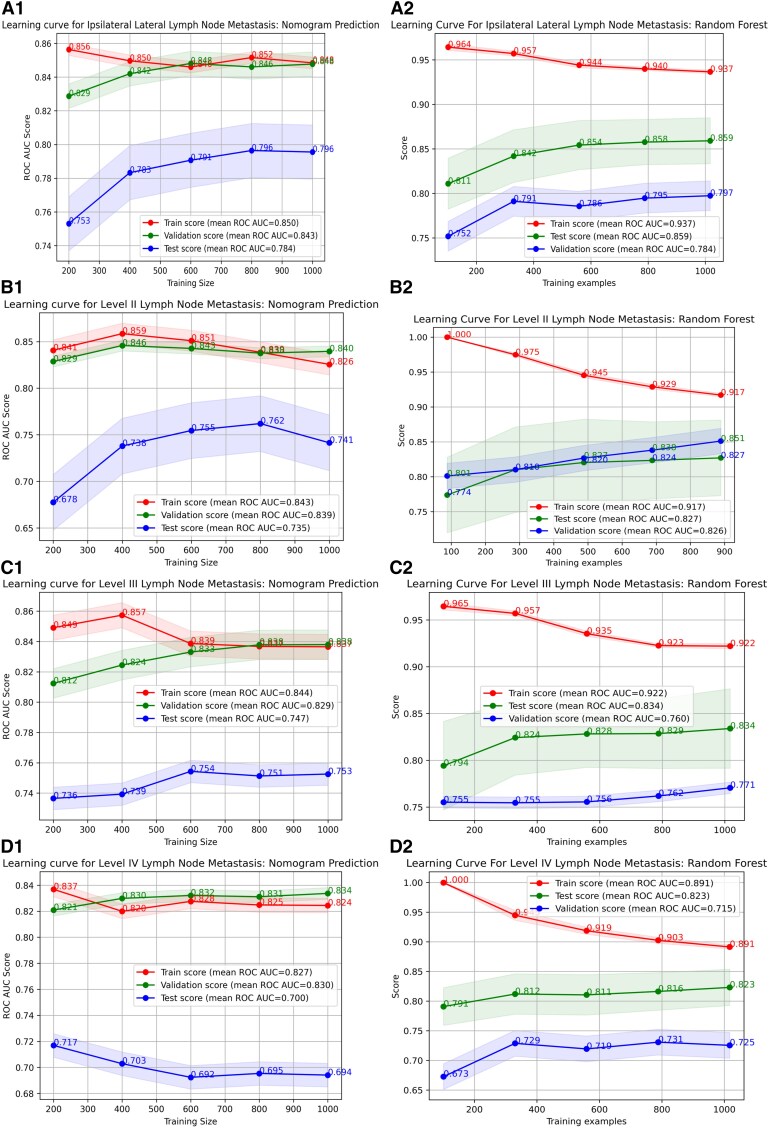
Comparison of the learning curves for the traditional nomogram prediction model and the optimal machine model (random forest) for predicting lateral lymph node metastasis: (A1) Learning curve of the nomogram prediction model for ipsilateral lateral lymph node metastasis. (A2) Learning curve of the random forest model for ipsilateral lateral lymph node metastasis. (B1) Learning curve of the nomogram prediction model for level II lymph node metastasis. (B2) Learning curve of the random forest model for level II lymph node metastasis. (C1) Learning curve of the nomogram prediction model for level III lymph node metastasis. (C2) Learning curve of the random forest model for level III lymph node metastasis. (D1) Learning curve of the nomogram prediction model for level IV lymph node metastasis. (D2) Learning curve of the random forest model for level IV lymph node metastasis.

**Figure 6.**
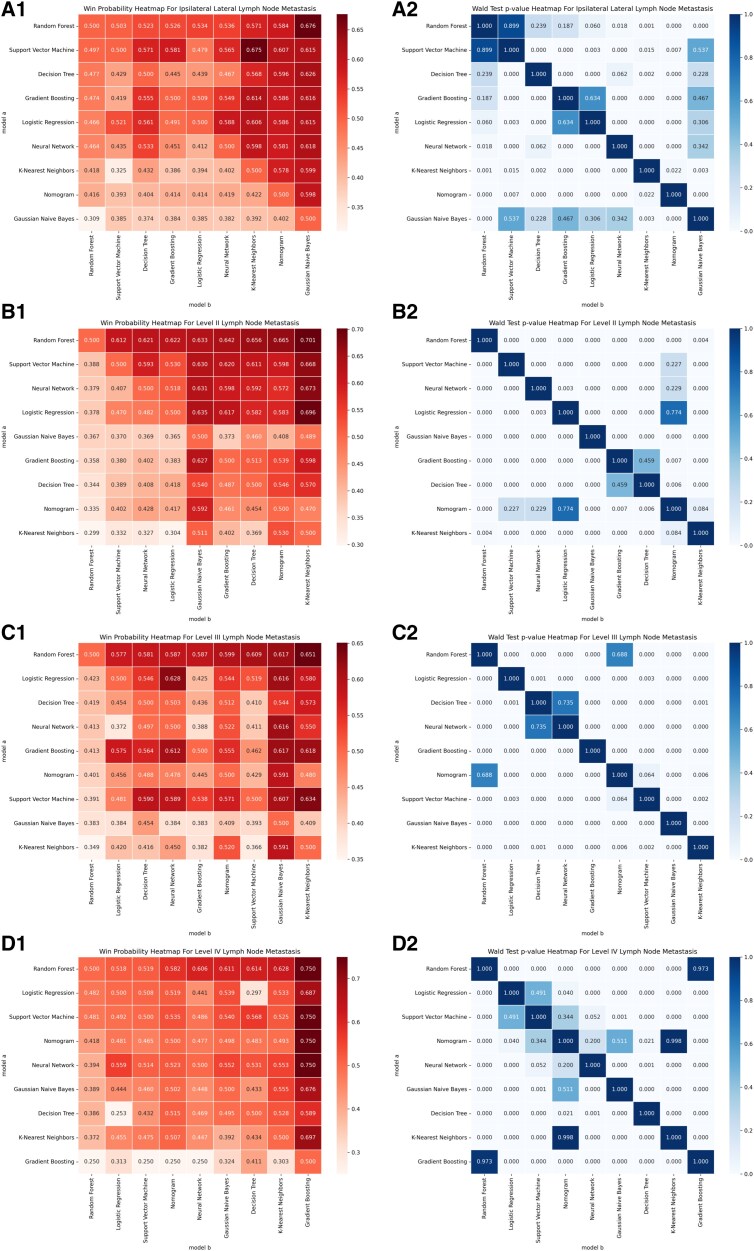
Comparison of win PMRA and Wald test *P*-values for the traditional nomogram and machine learning models in predicting lateral lymph node metastasis. (A1) Win probabilities estimated by PMRA for predicting ipsilateral lateral lymph node metastasis. (A2) Wald test *P*-values for predicting ipsilateral lateral lymph node metastasis. (B1) Win probabilities estimated by PMRA for predicting level II lymph node metastasis. (B2) Wald test *P*-values for predicting level II lymph node metastasis. (C1) Win probabilities estimated by PMRA for predicting level III lymph node metastasis. (C2) Wald test *P*-values for predicting level III lymph node metastasis. (D1) Win probabilities estimated by PMRA for predicting level IV lymph node metastasis. (D2) Wald test *P*-values for predicting level IV lymph node metastasis. Abbreviations: PMRA, probabilities estimated by probability-based ranking model approach.

**Figure 7.**
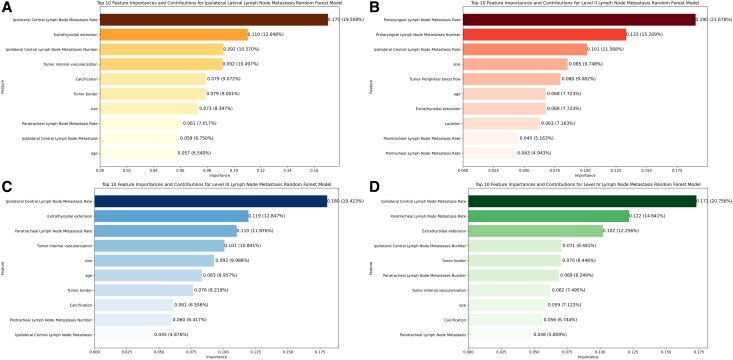
The importance values and contributions for the random forest predictive model of lateral lymph node metastasis. (A) Top 10 feature importance values and contributions bar chart for ipsilateral lateral lymph node metastasis. (B) Top 10 feature importance values and contributions bar chart for level II lymph node metastasis. (C) Top 10 feature importance values and contributions bar chart for level III lymph node metastasis. (D) Top 10 feature importance values and contributions bar chart for level IV lymph node metastasis.

To predict ipsilateral lateral LNM, 16 variables were selected using L1-norm feature selection, including tumor border, calcification, internal tumor vascularization, size, location, ETE, T staging, ipsilateral central LNM, recurrent laryngeal LNM, age, ipsilateral central LNM, ipsilateral central LNMN, pretracheal LNMN, paratracheal LNMR, and recurrent laryngeal LNMN. Detailed information is provided in Supplementary Material 2 (26). Using these variables, the optimal ML model was identified as the RF model. In terms of predictive accuracy, the RF model outperformed 7 other models, with AUC values of 0.858 in the test set and 0.799 in the validation set [Fig. 3 and Supplemental Figs.1 to 3 in Supplementary Material 3 (27)]. The DCA curve showed that the RF model had greater clinical utility compared to the other models [Fig. 4 and Supplemental Fig. 4 in Supplementary Material 3 (27)]. Additionally, the RF model's learning curve demonstrated the best fit, with mean AUC values of 0.859 for the test set and 0.784 for the validation set [Fig. 5 and Supplemental Fig. 5 in Supplementary Material 3 (27)]. The plotted calibration curve and precision-recall curve also yielded consistent results, as shown in Figs. 6 and 7 and Supplemental Figs. 6 and 7 in Supplementary Material 3 (27). The RF model was also the most effective in predicting LNM at levels II, III, and IV [see Supplementary Material 3 (27) for details].

### Comparison of the Optimal ML Prediction Model (RF) and the Traditional Prediction Model (Nomogram Prediction)

When comparing evaluation metrics, the RF model outperformed the other models in predicting ipsilateral LNM. Specifically, the RF model was significantly more accurate than the linear model in both the test set (0.773 vs 0.746) and the validation set (0.728 vs 0.685). The RF also demonstrated superior predictive capabilities, with a specificity and sensitivity on the test set of 0.984 and 0.757, respectively, and on the validation set, these values were 0.935 and 0.807, respectively. These values were all higher than those of the linear model: 0.752 and 0.740 (test set); 0.783 and 0.588, (validation set) (Tables 2 and 3). Moreover, ROC curve analysis revealed that the RF model had higher AUC values across all datasets: 0.934 (training set), 0.858 (test set), and 0.799 (validation set), compared to the nomogram's 0.86, 0.83, and 0.731, respectively (Fig. 3). The DCA curves further confirmed the superior clinical utility of the RF model, as its curve consistently outperformed the nomogram's curve (Fig. 4). The learning curve also showed higher mean AUC values for the RF model, especially in the training and test sets (Fig. 5). However, the AUC difference between the RF model's training (0.937) and validation (0.784) sets suggested potential overfitting, compared to the nomogram's AUCs (0.856 vs 0.767) [Supplementary Material 3 (27)]. Finally, based on the PMRA for predicting ipsilateral lateral LNM, the RF model had the highest probability of winning. support vector machine, decision tree, and gradient boosting models showed no significant differences from the RF model, though their probabilities of winning were all below 0.5. Logistic regression and neural network models performed slightly worse than the RF model, while K-nearest neighbor, nomogram, and GNB performed significantly worse, with GNB having the lowest probability of winning (0.309) (Table 4 and Fig. 6). A similar approach carried out for the other dependent variables revealed that the RF model also performed best in predicting LMN at levels II, III, and IV [Supplementary Material 3 (27)].

**Table 3.** Evaluation metrics of the optimal machine learning model (random forest) in train test, and validation sets for ipsilateral lateral, level II, level III, and level IV lymph node metastasis

Regions	Accuracy	AUC	Specificity	Sensitivity	NPV	PPV	F1 Score	FPR	Lift	Brier score	Kappa
Testing set (internal validation set)											
Ipsilateral lateral LNM	0.773	0.858	0.984	0.757	0.78	0.935	0.755	0.2	1.797	0.154	0.544
Level II LNM	0.825	0.848	0.966	0.784	0.915	0.7	0.537	0.028	2.731	0.131	0.413
Level III LNM	0.781	0.831	0.895	0.719	0.847	0.693	0.657	0.105	2.148	0.154	0.489
Level IV LNM	0.798	0.831	0.97	0.691	0.879	0.667	0.566	0.03	2.403	0.139	0.436
Validation set (external validation set)											
Ipsilateral lateral LNM	0.728	0.799	0.935	0.807	0.779	0.837	0.744	0.165	1.604	0.188	0.458
Level II LNM	0.809	0.813	0.997	0.591	0.877	0.875	0.462	0.003	2.545	0.135	0.378
Level III LNM	0.724	0.772	0.769	0.644	0.795	0.609	0.626	0.231	1.753	0.188	0.408
Level IV LNM	0.733	0.722	0.956	0.377	0.878	0.529	0.414	0.148	1.818	0.176	0.243

Abbreviations: AUC, area under the curve; FPR, false positive rate; LNM, lymph node metastasis; NPV, negative predictive value; PPV, positive predictive value.

**Table 4.** Ranking by probability-based ranking model approach for predicting ipsilateral lateral lymph node metastasis

	Models	Probabilities of win against top model	Wald *P*-value
1	Random forest	/	/
2	Support vector machine	0.497	.899
3	Decision tree	0.477	.239
4	Gradient boosting	0.474	.187
5	Logistic regression	0.466	.06
6	Neural network	0.464	.018
7	K-nearest neighbors	0.418	.001
8	Nomogram	0.416	<.001
9	Gaussian naive bayes	0.309	<.001

### Variable Importance and Network Calculator

**Figure 8.**
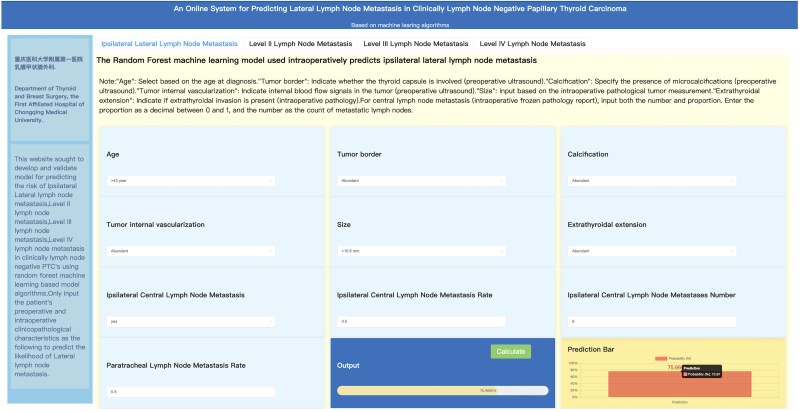
Neural network calculator for lateral lymph node metastasis. The web calculator interface shown in the figure tests a patient over 43 years old with an unclear tumor boundary, microcalcification, internal blood flow signals, a tumor diameter over 10.5 mm, intraoperative pathological evidence of tumor invasion, and 6/12 paratracheal lymph node metastases. The calculated ipsilateral lateral lymph node metastasis rate is 75.97%.

The top 10 most important variables for predicting ipsilateral lateral LNM, ranked by importance value and contribution percentage, included ipsilateral central LNMR (importance value of 0.170, contribution of 19.508%), extrathyroidal extension (0.110, 12.648%), ipsilateral central LNMN (0.092, 10.497%), calcification (0.079, 9.072%), tumor border (0.079, 9.001%), size (0.073, 8.397%), paratracheal LNMR (0.061, 7.017%), ipsilateral central LNM (0.059, 6.750%), and age (0.057, 6.540%). As shown in Supplementary Material 4 (28), the model incorporating both preoperative and intraoperative features demonstrated superior performance compared to using preoperative features alone. Additionally, as shown in Supplementary Material 5 (29), we conducted a comparative analysis between the RF model using only these top 10 variables and the nomogram model. The results demonstrated that the simplified RF model outperformed the nomogram in training, testing, and validation sets in terms of both area under the ROC curve (AUC: 0.934 vs 0.86, 0.858 vs 0.83, and 0.794 vs 0.766, respectively) and accuracy (0.856 vs 0.78, 0.763 vs 0.746, and 0.712 vs 0.685, respectively). Based on these findings, we have developed a web-based calculator based on these variables (Fig. 8). The calculator's output utilizes the RF model, which incorporates the top 10 variables and their feature importance values to predict LNM likelihood. For example, an output of 75.97% indicates a 75.97% likelihood of ipsilateral lateral LNM. The web-based calculator provides real-time predictions based on the input data and is designed to be user-friendly for clinicians. The website address is http://121.41.36.155:9001/static/html/index.html.

## Discussion

### Selection and Significance of the Optimal Model

Because of the ongoing controversy surrounding the surgical management of cN0-PTC (7-9), accurate preoperative prediction of the lymph node status of PTC patients is crucial for the precise treatment of cN0-PTC patients. Assessing lymph node status is crucial in determining the extent of selective LND (13-15), making the development of reliable tools for lymph node evaluation essential. In this multicenter retrospective study, we developed a comprehensive model integrating clinical, ultrasound, and intraoperative frozen pathology data to predict ipsilateral lateral LNM and subregion LNM in patients with PTC. This model aims to assist in decision-making about when and which regions to include in LND.

The RF model demonstrated the highest performance in predicting LNM in the ipsilateral lateral region and subregions. In the internal validation set, the AUC values for ipsilateral lateral and level II, III, and IV LNM were 0.858, 0.848, 0.831, and 0.831, respectively. In the external validation set, the AUC values for ipsilateral lateral and level II, III, and IV LNM were 0.799, 0.813, 0.772, and 0.722 respectively (27). These high AUC values reflect the RF model's strong discriminative ability and accuracy in predicting LNM. Beyond assessing predictive accuracy, we evaluated clinical utility using DCA to compare the practical value of our model. The RF model proved effective for determining whether to perform ipsilateral lateral LND and subregion LND. Our comparison of the RF model with traditional nomogram models, using ROC curves, DCA curves, learning curves, a win rate probability heatmap based on the PMRA, and a *P*-value heatmap of the Wald test, highlights the superior predictive capability and clinical applicability of the RF model (27).

This personalized model meets the requirements of precision medicine and helps surgeons select their surgical strategy. Previous studies, such as those by Zhu et al and Wang et al, also developed predictive models for cervical LNM using ML algorithms and clinical features (19, 20). Zhu et al (20) employed a RF model with an AUC of 0.812, an accuracy of 0.734, a specificity of 0.788, and a sensitivity of 0.684 in internal validation. Wang et al (19) compared a deep learning convolutional neural network with a traditional nomogram, achieving an AUC of 0.78, specificity of 0.82, and sensitivity of 0.62. Our study, utilizing only preoperative features, achieved an AUC of 0.813, accuracy of 0.74, specificity of 0.839, and sensitivity of 0.63 (28), demonstrating stronger predictive performance than Wang et al's study and similar performance to Zhu et al's (19, 20).

Moreover, while previous studies relied solely on clinical and ultrasound data and used bootstrapping for internal validation—potentially leading to overfitting—our model incorporates both preoperative and intraoperative features, providing significantly improved performance. The RF model for predicting ipsilateral lateral LNM in our test set achieved an accuracy of 0.773, AUC of 0.858, specificity of 0.984, and sensitivity of 0.757, surpassing the performance of preoperative models (28). Additionally, our multicenter study included independent internal and external validation sets and exhibited excellent predictive performance across both sets of datasets. Compared to traditional nomograms (18, 20), our model, which is based on evidence from intraoperative frozen pathology, combines valuable predictive information on ipsilateral central LNM and subgroups of central LNM, clinical risk factors, and ultrasound features, which can be obtained rapidly and accurately during surgery. This combination model is a novel approach for predicting lateral LNM (29).

### Analysis of the Predictive Value of Factors Affecting Ipsilateral Lateral LNM and Subregions of Ipsilateral Lateral LNM

This study successfully identified significant parameters that influence the prediction of ipsilateral lateral LNM and its subregions. Among the top 10 key parameters, the presence of central LNM emerged as the most significant predictor for ipsilateral lateral LNM. The importance value for the ratio of ipsilateral central LNMR was 0.17. Additionally, in the multivariable analysis, ipsilateral central LNM had the highest OR of 4.915 (*P* < .001). Central LNM was also a critical factor for predicting subregions of ipsilateral lateral LNM. It was the most important positive predictive parameter for both level III and level IV LNM, with importance values of 0.18 and 0.173, respectively. The OR values for ipsilateral central LNM in these levels were 2.849 and 2.907, respectively (25). For level II LNM, ipsilateral central LNMR was the third most influential factor with an importance value of 0.101, and the OR for ipsilateral central LNM was 1.988 (*P* < .001) (25). These results corroborate the significant predictive value of central LNM for lateral LNM, aligning with findings from previous studies by our research group and others (30-32). For instance, Feng et al identified the ratio of central LNM as the most critical factor for lateral LNM (33). Lee et al recommended performing prophylactic lateral LND when central LNMN is ≥2 (34). Huang et al reported that central LNMN ≥ 3 was an independent risk factor for lateral LNM (9). Machens et al reported that when the central LNMN was 5 or more, the lateral LNMR increased from 45% to 69% to 100% (35). Furthermore, ETE has been widely recognized as a significant independent risk factor for lateral LNM in PTC (15, 36). In our study, ETE was the second most important predictor for lateral LNM, with an importance value of 0.11 (OR = 3.692, *P* < .001), and demonstrated high predictive values across subregions, with importance values of 0.119 for level III LNM and 0.102 for level IV LNM. Our findings are consistent with previous research and clinical knowledge, reinforcing the accuracy of the RF model (14, 15, 33-35).

The prelaryngeal LNM status is the most important parameter among the top 10 key parameters in level II LNM, with the highest importance value of 0.19 and an OR of 4.263 (*P* < .001). The importance value for prelaryngeal LNMN is 0.133, which may be related to the lymphatic drainage pathway of the thyroid gland (37). This study further supports previous findings (38, 39), noting that prelaryngeal lymph nodes can serve as sentinel lymph nodes for level II LNM due to their anatomical position and role as the first station for metastasis in the upper part of the tumor (40-43). Additionally, the tumor's location in the upper portion had a higher positive predictive value for level II LNM, with an importance value of 0.063 (OR = 4.284, *P* < .001). These findings further support the view that LNM in PTC is traceable and highly consistent with the thyroid drainage pathway. While prelaryngeal lymph nodes alone cannot provide a complete picture of regional LNM, they are significant for identifying level II LNM. Among the subregions with level II LNM, the transfer rate is lowest in each side region (44, 45), The low transfer rate of level II LNM—21.341% in our study—highlights the challenge for surgeons: identifying this small subset of patients while avoiding complications from excessive dissection in the majority who do not have metastasis (45). This study addresses this problem and emphasizes the importance of level II LNM, especially in patients with prelaryngeal LNM in the upper portion and a high number and proportion of metastases. Prophylactic level II LND should be considered if necessary.

The factors influencing level III LNM are highly similar to those influencing ipsilateral lateral LNM. This is partly because the ipsilateral central zone has the highest proportion among the subregions of the lateral zone, accounting for 32.5%. Additionally, the complexity of thyroid lymphatic drainage pathways impacts level III LNM. These pathways are extensive and interconnected (37), which explains why tumor location was not a significant factor in predicting level III LNM, as reflected by its exclusion in univariate analysis with a *P*-value of .651. This finding is consistent with previous research findings (46, 47). The proportions of pretracheal LNM and paratracheal LNM within the subregions of the central zone are important factors for predicting level III LNM, with slightly lower importance values than those of ipsilateral central LNMR. However, the predictive value of prelaryngeal LNM and pretracheal LNM for level III LNM was not high in this study. This result is consistent with previous research findings (38, 48).

Among the top 10 parameters for level IV LNM, the ipsilateral central LNMR was again a key factor, with an importance value of 0.173. Another critical predictor was the presence of paratracheal LNM, with an importance value of 0.122 (OR = 2.096, *P* < .001). LNMR is the most important factor influencing level IV LNM. In terms of lymphatic drainage pathways, level IV LNM occurs along the descending lymphatic pathways, following the distribution of the inferior thyroid artery and extending through paratracheal lymph nodes to the venous angle (37). This pathway further supports the view that patients with paratracheal LNM are prone to metastasis in region IV. This observation is supported by studies such as Kim et al.’s research, which found a strong correlation between paratracheal and recurrent laryngeal LNM and lateral LNM (49). This finding is consistent with the research conducted by Zhou et al (48, 50).

One of the major advantages of this study is the development of a powerful yet user -riendly predictive model. By incorporating the top 10 feature variables identified as most important through ML, we achieved a balance between model performance and clinical applicability. Importantly, we have integrated insights from another study conducted by our research center, which focuses on paratracheal lymph node metastasis in cN0 papillary thyroid carcinoma (51). This complementary research highlights the predictive value of central lymph node metastasis features, providing additional context to our findings and enhancing the utility of our model in surgical decision-making.

To facilitate clinical application, we developed an online tool (http://121.41.36.155:9001/static/html/index.html) based on the RF model using the top 10 feature variables (29). For example, as shown in Fig. 8, there is a patient over 43 years old with an unclear tumor boundary, calcification, internal blood flow signals, a tumor diameter over 10.5 mm, intraoperative pathological evidence of tumor invasion, and 6/12 paratracheal LNM. The calculated lateral LNMR is 75.97%. This suggests that selective prophylactic lateral LND may be considered, enabling more tailored surgical strategies. By incorporating the findings of both studies, this tool aims to provide clinicians with a more comprehensive and precise approach to managing cN0 papillary thyroid carcinoma.

### Limitations of This Study

This study has several limitations that should be acknowledged. First, the criteria for lateral compartment dissection in our study were predetermined, which introduced selection bias, particularly toward patients with specific clinical features and intraoperative findings. This selection bias is further reflected in the statistical results, which showed smaller tumor sizes and lower BMI values. In the context of our country, there is limited understanding of the malignant progression of PTC, leading to a preference for proactive treatment over follow-up. This behavior, driven by health examinations and a predominance of younger patients, has introduced skewness into the data. Although we applied data augmentation techniques and numerical data correction to mitigate this bias, it was not entirely eliminated. Future research should aim to address this issue through more representative sampling. Additionally, while we achieved high AUC values in internal and external validations across multiple centers, the validation results should be interpreted with caution. The observed lower performance in the validation set may be partially due to differences in the age of the data and potential clinical scoring drift over time, reflecting discrepancies between the training and validation populations. This limitation is beyond our control and suggests that further prospective testing is needed to establish the clinical applicability of the model. Additionally, the learning curves indicate that the RF prediction model for ipsilateral and level II, III, and IV LNM had higher average AUC values in the test and validation sets compared to traditional nomograms, demonstrating better predictive performance. However, the model exhibited poor convergence and generalization, suggesting some degree of overfitting. We attempted to mitigate overfitting by optimizing feature engineering and using 6 ML algorithms to select the best features, coupled with 10-fold cross-validation to choose optimal parameters for model training. This limitation might be related to the small sample size in the training set. Future studies should aim to increase the sample size and incorporate more robust model optimization strategies to enhance generalizability. Third, on the basis of our study results, we have preliminarily developed a simple tool for predicting the probability of lateral LNM. However, determining the practical value of this tool requires further prospective studies and long-term follow-up, incorporating genomics and radiomics. Lastly, the manual input of parameters during model construction may have led to overlooked or unknown pathological features, potentially missing hidden relationships. To mitigate this risk, we ensured consistent data analysis to enhance model accuracy. Nonetheless, prospective validation and larger studies are essential to confirm the model's robustness in clinical practice. With successful validation, we believe the RF model has significant potential for guiding individualized lateral neck dissection in PTC patients.

## Conclusion

Using preoperative variables and intraoperative frozen pathology features, we developed and then internally and externally validated various ML predictive models, comparing them with traditional prediction models. We identified the optimal predictive model, namely, the RF algorithm, for individualized prediction of ipsilateral lateral and subregional lateral LNM in cN0-PTC patients. ML-based predictive models accurately identify patients at high risk of lateral LNM and specific subregional involvement. The accompanying online risk calculator serves as a user-friendly tool for clinicians to make precise surgical decisions. In the future, our goal is to further integrate imaging, molecular, and genetic data to enhance the performance of our model in the field of personalized medicine, and further validation through studies involving a broader population is needed.

## Data Availability

Original data generated and analyzed during this study are included in this published article or in the data repositories listed in References (52).

## References

[1] Megwalu UC, Moon PK. Thyroid cancer incidence and mortality trends in the United States: 2000-2018. Thyroid. 2022;32(5):560‐570. 35132899 10.1089/thy.2021.0662

[2] Filetti S, Durante C, Hartl D, et al Thyroid cancer: ESMO Clinical Practice Guidelines for diagnosis, treatment and follow-updagger. Ann Oncol. 2019;30(12):1856‐1883. 31549998 10.1093/annonc/mdz400

[3] Jang SW, Park JH, Kwon HJ, Yoon JH. Optimal cutoff values of primary tumour size to better predict long-term outcomes in patients with papillary thyroid carcinoma undergoing total thyroidectomy: a preliminary study using restricted cubic spline analysis. Clin Endocrinol (Oxf). 2022;96(6):888‐895. 34908183 10.1111/cen.14657

[4] Dadafarin S, Rodriguez TC, Carnazza MA, Tiwari RK, Moscatello A, Geliebter J. MEG3 expression indicates lymph node metastasis and presence of cancer-associated fibroblasts in papillary thyroid cancer. Cells. 2022;11(19):3181. 36231143 10.3390/cells11193181 PMC9562881

[5] Haugen BR, Alexander EK, Bible KC, et al 2015 American thyroid association management guidelines for adult patients with thyroid nodules and differentiated thyroid cancer: the American thyroid association guidelines task force on thyroid nodules and differentiated thyroid cancer. Thyroid. 2016;26(1):1‐133. 10.1089/thy.2015.0020 PMC4739132 26462967

[6] Zhang X, Li JG, Zhang SZ, Chen G. Comparison of indocyanine green and carbon nanoparticles in endoscopic techniques for central lymph nodes dissection in patients with papillary thyroid cancer. Surg Endosc. 2020;34(12):5354‐5359. 31907662 10.1007/s00464-019-07326-4

[7] Ahn JH, Kwak JH, Yoon SG, et al A prospective randomized controlled trial to assess the efficacy and safety of prophylactic central compartment lymph node dissection in papillary thyroid carcinoma. Surgery. 2022;171(1):182‐189. 34391573 10.1016/j.surg.2021.03.071

[8] Huang J, Li Z, Zhong Q, et al Developing and validating a multivariable machine learning model for the preoperative prediction of lateral lymph node metastasis of papillary thyroid cancer. Gland Surg. 2023;12(1):101‐109. 36761483 10.21037/gs-22-741 PMC9906091

[9] Huang H, Xu S, Ni S, Wang X, Liu S. A nomogram for predicting lateral lymph node metastasis in cN0 unifocal papillary thyroid microcarcinoma. BMC Cancer. 2023;23(1):718. 37528388 10.1186/s12885-023-11219-0 PMC10391989

[10] Lee DW, Ji YB, Sung ES, et al Roles of ultrasonography and computed tomography in the surgical management of cervical lymph node metastases in papillary thyroid carcinoma. Eur J Surg Oncol. 2013;39(2):191‐196. 22863305 10.1016/j.ejso.2012.07.119

[11] Zhao H, Li H. Meta-analysis of ultrasound for cervical lymph nodes in papillary thyroid cancer: diagnosis of central and lateral compartment nodal metastases. Eur J Radiol. 2019;112:14‐21. 30777203 10.1016/j.ejrad.2019.01.006

[12] Alabousi M, Alabousi A, Adham S, et al Diagnostic test accuracy of ultrasonography vs computed tomography for papillary thyroid cancer cervical lymph node metastasis: a systematic review and meta-analysis. JAMA Otolaryngol Head Neck Surg. 2022;148(2):107‐118. 34817554 10.1001/jamaoto.2021.3387 PMC8613701

[13] Scherl S, Mehra S, Clain J, et al The effect of surgeon experience on the detection of metastatic lymph nodes in the central compartment and the pathologic features of clinically unapparent metastatic lymph nodes: what are we missing when we don't perform a prophylactic dissection of central compartment lymph nodes in papillary thyroid cancer? Thyroid. 2014;24(8):1282‐1288. 24787362 10.1089/thy.2013.0600

[14] Patron V, Hitier M, Bedfert C, Metreau A, Dugue A, Jegoux F. Predictive factors for lateral occult lymph node metastasis in papillary thyroid carcinoma. Eur Arch Otorhinolaryngol. 2013;270(7):2095‐2100. 23238703 10.1007/s00405-012-2305-z

[15] Ma B, Wang Y, Yang S, Ji Q. Predictive factors for central lymph node metastasis in patients with cN0 papillary thyroid carcinoma: a systematic review and meta-analysis. Int J Surg. 2016;28:153‐161. 26944586 10.1016/j.ijsu.2016.02.093

[16] Raffaelli M, Crea D, Sessa C, et al Can intraoperative frozen section influence the extension of central neck dissection in cN0 papillary thyroid carcinoma? Langenbecks Arch Surg. 2013;398(3):383‐388. 23207498 10.1007/s00423-012-1036-3

[17] Kim MJ, Kim HJ, Park CS, Kim BW. Frozen section analysis of central lymph nodes in papillary thyroid cancer: the significance in determining the extent of surgery. Gland Surg. 2022;11(4):640‐650. 35531106 10.21037/gs-22-15 PMC9068541

[18] Shu X, Tang L, Hu D, et al Prediction model of pathologic central lymph node negativity in cN0 papillary thyroid carcinoma. Front Oncol. 2021;11:727984. 34646771 10.3389/fonc.2021.727984 PMC8503674

[19] Wang Z, Qu L, Chen Q, et al Deep learning-based multifeature integration robustly predicts central lymph node metastasis in papillary thyroid cancer. BMC Cancer. 2023;23(1):128. 36750791 10.1186/s12885-023-10598-8 PMC9906958

[20] Zhu H, Yu B, Li Y, et al Models of ultrasonic radiomics and clinical characters for lymph node metastasis assessment in thyroid cancer: a retrospective study. PeerJ. 2023;11:e14546. 36650830 10.7717/peerj.14546 PMC9840861

[21] Zou Y, Shi Y, Sun F, et al Extreme gradient boosting model to assess risk of central cervical lymph node metastasis in patients with papillary thyroid carcinoma: individual prediction using SHapley Additive exPlanations. Comput Methods Programs Biomed. 2022;225:107038. 35930861 10.1016/j.cmpb.2022.107038

[22] Agyekum EA, Ren YZ, Wang X, et al Evaluation of cervical lymph node metastasis in papillary thyroid carcinoma using clinical-ultrasound radiomic machine learning-based model. Cancers (Basel). 2022;14(21):5266. 36358685 10.3390/cancers14215266 PMC9655605

[23] Yu J, Deng Y, Liu T, et al Lymph node metastasis prediction of papillary thyroid carcinoma based on transfer learning radiomics. Nat Commun. 2020;11(1):4807. 32968067 10.1038/s41467-020-18497-3 PMC7511309

[24] Gajda S, Chlebus M. A probability-based models ranking approach: an alternative method of machine-learning model performance assessment. Sensors (Basel). 2022;22(17):6361. 36080820 10.3390/s22176361 PMC9460558

[25] Zhou J, Ren JH, Su XL. Supplementary material 1 from: the First Affiliated Hospital of Chongqing Medical University in China: Generic Digital Repository-Figshare 2024. 10.6084/m9.figshare.26317225. Deposited 17 July 2024. Accessed 15 September 2024.

[26] Zhou J, Ren JH, Su XL. Supplementary material 2 from: the First Affiliated Hospital of Chongqing Medical University in China: Generic Digital Repository-Figshare 2024. 10.6084/m9.figshare.26317405. Deposited 17 July 2024. Accessed 20 July 2024.

[27] Zhou J, Ren JH, Su XL. Supplementary material 3 from: the First Affiliated Hospital of Chongqing Medical University in China: Generic Digital Repository-Figshare 2024. 10.6084/m9.figshare.26317480. Deposited 17 July 2024. Accessed 15 September 2024.

[28] Zhou J, Ren JH, Su XL. Supplementary material 4 from: the First Affiliated Hospital of Chongqing Medical University in China: Generic Digital Repository-Figshare 2024. 10.6084/m9.figshare.26319661. Deposited 17 July 2024. Accessed 20 July 2024.

[29] Zhou J, Ren JH, Su XL. Supplementary material 5 from: the First Affiliated Hospital of Chongqing Medical University in China: Generic Digital Repository-Figshare 2024. 10.6084/m9.figshare.27021622. Deposited 15 September 2024. Accessed 15 September 2024.

[30] Hu D, Zhou J, He W, et al Risk factors of lateral lymph node metastasis in cN0 papillary thyroid carcinoma. World J Surg Oncol. 2018;16(1):30. 29439716 10.1186/s12957-018-1336-3 PMC5811970

[31] Wang Y, Deng C, Shu X, et al Risk factors and a prediction model of lateral lymph node metastasis in CN0 papillary thyroid carcinoma patients with 1-2 central lymph node metastases. Front Endocrinol (Lausanne). 2021;12:716728. 34721289 10.3389/fendo.2021.716728 PMC8555630

[32] Dou Y, Chen Y, Hu D, Xiong W, Xiao Q, Su X. Development and validation of web-based nomograms for predicting lateral lymph node metastasis in patients with papillary thyroid carcinoma. Gland Surg. 2020;9(2):172‐182. 32420240 10.21037/gs.2020.01.11 PMC7225478

[33] Feng JW, Ye J, Qi GF, et al A comparative analysis of eight machine learning models for the prediction of lateral lymph node metastasis in patients with papillary thyroid carcinoma. Front Endocrinol (Lausanne). 2022;13:1004913. 36387877 10.3389/fendo.2022.1004913 PMC9651942

[34] Lee YS, Lim YS, Lee JC, Wang SG, Kim IJ, Lee BJ. Clinical implication of the number of central lymph node metastasis in papillary thyroid carcinoma: preliminary report. World J Surg. 2010;34(11):2558‐2563. 20703463 10.1007/s00268-010-0749-0

[35] Machens A, Hauptmann S, Dralle H. Lymph node dissection in the lateral neck for completion in central node-positive papillary thyroid cancer. Surgery. 2009;145(2):176‐181. 19167972 10.1016/j.surg.2008.09.003

[36] Song RY, Kim HS, Kang KH. Minimal extrathyroidal extension is associated with lymph node metastasis in single papillary thyroid microcarcinoma: a retrospective analysis of 814 patients. World J Surg Oncol. 2022;20(1):170. 35643530 10.1186/s12957-022-02629-8 PMC9148524

[37] Likhterov I, Reis LL, Urken ML. Central compartment management in patients with papillary thyroid cancer presenting with metastatic disease to the lateral neck: Anatomic pathways of lymphatic spread. Head Neck. 2017;39(5):853‐859. 28252836 10.1002/hed.24568

[38] Wang B, Zhu CR, Liu H, Yao XM, Wu J. Relationship between pretracheal and/or prelaryngeal lymph node metastasis and paratracheal and lateral lymph node metastasis of papillary thyroid carcinoma: a meta-analysis. Front Oncol. 2022;12:950047. 36212418 10.3389/fonc.2022.950047 PMC9543714

[39] Yan Y, Wang Y, Liu N, et al Predictive value of the Delphian lymph node in cervical lymph node metastasis of papillary thyroid carcinoma. Eur J Surg Oncol. 2021;47(7):1727‐1733. 33632590 10.1016/j.ejso.2021.02.010

[40] Kim DH, Kim SW, Hwang SH. Predictive value of delphian lymph node metastasis in the thyroid cancer. Laryngoscope. 2021;131(9):1990‐1996. 33493364 10.1002/lary.29426

[41] Yan XQ, Ma ZS, Zhang ZZ, et al The utility of sentinel lymph node biopsy in the lateral neck in papillary thyroid carcinoma. Front Endocrinol (Lausanne). 2022;13:937870. 35957824 10.3389/fendo.2022.937870 PMC9357979

[42] Song Y, Xu G, Wang T, Zhang Y, Zhang B. Indications of superselective neck dissection in patients with lateral node metastasis of papillary thyroid carcinoma. Otolaryngol Head Neck Surg. 2022;166(5):832‐839. 34488520 10.1177/01945998211038318

[43] Chai YJ, Kim SJ, Choi JY, Koo do H, Lee KE, Youn YK. Papillary thyroid carcinoma located in the isthmus or upper third is associated with Delphian lymph node metastasis. World J Surg. 2014;38(6):1306‐1311. 24366273 10.1007/s00268-013-2406-x

[44] Lim YS, Lee JC, Lee YS, et al Lateral cervical lymph node metastases from papillary thyroid carcinoma: predictive factors of nodal metastasis. Surgery. 2011;150(1):116‐121. 21507446 10.1016/j.surg.2011.02.003

[45] Lv T, Ma WL, Tan Z, et al Level II lateral neck dissection for papillary thyroid carcinoma: a retrospective cohort study. Asian J Surg. 2023;46(10):4290‐4295. 37085417 10.1016/j.asjsur.2023.04.003

[46] Song L, Zhou J, Chen W, et al Lymph node metastasis between the sternocleidomastoid and sternohyoid muscle in papillary thyroid carcinoma patients: a prospective study at multiple centers. Asian J Surg. 2021;44(8):1043‐1049. 33581944 10.1016/j.asjsur.2021.01.005

[47] Liu WQ, Yang JY, Wang XH, Cai W, Li F. Analysis of factors influencing cervical lymph node metastasis of papillary thyroid carcinoma at each lateral level. BMC Surg. 2022;22(1):228. 35701785 10.1186/s12893-022-01678-w PMC9199251

[48] Zhou M, Duan Y, Ye B, et al Pattern and predictive factors of metastasis in lymph nodes posterior to the right recurrent laryngeal nerve in papillary thyroid carcinoma. Front Endocrinol (Lausanne). 2022;13:914946. 35923627 10.3389/fendo.2022.914946 PMC9339603

[49] Kim D, Kwon HK, Shin SC, et al Right posterior paratracheal lymph nodes metastasis is one of the predictive factors in right-sided papillary thyroid carcinoma. Surgery. 2019;166(6):1154‐1159. 31444006 10.1016/j.surg.2019.06.024

[50] Zou M, Wang YH, Dong YF, Lai XJ, Li JC. Clinical and sonographic features for the preoperative prediction of lymph nodes posterior to the right recurrent laryngeal nerve metastasis in patients with papillary thyroid carcinoma. J Endocrinol Invest. 2020;43(10):1511‐1517. 32253729 10.1007/s40618-020-01238-0

[51] Chun L, Wang D, He L, et al Explainable machine learning model for predicting paratracheal lymph node metastasis in cN0 papillary thyroid cancer. Sci Rep. 2024;14(1):22361. 39333646 10.1038/s41598-024-73837-3 PMC11436978

[52] Zhou J, Ren JH, Su XL. Data from: the First Affiliated Hospital of Chongqing Medical University in China:Generic Digital Repository-Figshare 2024. 10.6084/m9.figshare.26317171. Deposited 17 July 2024. Accessed 18 July 2024.

